# Giant Gyroid and Templates from High-Molecular-Weight Block Copolymer Self-assembly

**DOI:** 10.1038/srep36326

**Published:** 2016-11-03

**Authors:** Sungmin Park, Yeongsik Kim, Hyungju Ahn, Jong Hak Kim, Pil J. Yoo, Du Yeol Ryu

**Affiliations:** 1 Department of Chemical and Biomolecular Engineering, Yonsei University, Seoul 03722, Korea; 2 Pohang Accelerator Laboratory, Pohang University of Science and Technology, Pohang 37673, Korea; 3 School of Chemical Engineering and SKKU Advanced Institute of Nanotechnology (SAINT), Sungkyunkwan University (SKKU), Suwon 16419, Korea

## Abstract

We present a feasible approach to the direct development of three-dimensionally (3D) bicontinuous gyroid (GYR) nanostructure in high-molecular-weight, composition-controlled polystyrene-*b*-poly(methyl methacrylate) (PS-*b*-PMMA) films. The use of a neutral solvent vapor to elaborately control the swelling of block copolymer (BCP) films is essential to generate a direct pathway to GYR (or giant GYR) structure through a hexagonal (HEX) cylindrical morphology in the same material, because the thermal ordering of highly entangled BCP imposes the limit on the chain mobility. Along with the improved mechanical strength arising from the high molecular weight property of the polymers, the structural integrity and overall excellence of a large-scale GYR morphology were confirmed by the results of membrane performance, which showed greater permeability through the nanoporous GYR structure up to by a factor of three than that through the HEX structure. Moreover, a 3D nanoporous GYR template was applied to an affordable material to reproduce an inverse skeletal replica of the GYR structure with its structure being uniformly interconnected. This simple approach to the GYR template, owing to its structural tunability in a controlled composition of BCP, is anticipated to be applicable to a wide range of materialization for practical systems.

Materialization of well-organized, uniform nanostructures has currently been a challenge to meet the increasing demands for these materials in high-performance and high-precision applications such as high-density data storage^1^,^2^, bio-sensors^3^,^4^, water filtration^5^,^6^,^7^, controlled drug release^8^,^9^, and nanoscale replication^10^,^11^,^12^. Recently, much effort in modern nanotechnology has been dedicated to developing and achieving nanostructured materials that impart highly beneficial physicochemical properties. One of the appealing candidate methods for producing nanoscopic arrays and templates could be the directed self-assembly of block copolymers (BCPs), which have attracted extensive attention on account of its salient ability to produce nanoscopically ordered morphologies, like lamellae, cylinders, spheres and bicontinuously interconnected gyroids^13^,^14^,^15^,^16^.

Although the BCPs are essentially soft materials, in particular with regards to their mechanical stability, various strategies have been suggested to improve their structural properties such as by modifying chemical structures, fashioning them into diverse morphologies, and modifying the processes to produce these materials. A basic approach to enhance their mechanical strength is to utilize well-developed nanostructures from high-molecular-weight BCPs that ensure larger feature sizes in microphase-separated morphologies^17^,^18^. Yet, the ordering of long polymer chains is strongly unfavored by their limited chain mobility because a highly entangled conformation deteriorates the degree of translational order, hence the largely periodic structures of the BCPs have been a challenging task. Intriguingly, the chain mobility of high- and ultrahigh-molecular-weight BCPs could readily be accelerated by applying a solvent vapor annealing (SVA) process, and the long-range ordered morphologies have typically been regulated by annealing them for an appropriate period of time with a neutral solvent^19^,^20^. This SVA process is greatly favorable with speeding up the ordering rate and lowering the local energy barriers associated with defect annihilation. Besides, Thomas *et al.* reported highly oriented microdomain patterns of high-molecular-weight lamella- or cylinder-forming BCPs over large areas via directional solidification of a solvent, where the large-size microdomains were optically responsive as photonic bandgaps in the visible or infrared wavelengths^21^. Likewise, brush-type BCPs were synthesized by Grubbs *et al.*, and they showed large lamellar periods upon self-assembly^22^,^23^. The brush-type BCP chain architecture forms densely grafted side chains along the main chains, and the side chains with the different chemical identity are generally less entangled and radially stretched from the mainchains, eventually leading to a greater chain mobility for ordering of high-molecular-weight BCPs.

Meanwhile, the formation of three-dimensional (3D) textures from BCP self-assembly has been studied in the pursuit of developing new transport characteristics owing to the structurally interconnected pathways found in these materials. Wiesner and coworkers proposed various approaches to create porous and hierarchical structures using self-assembled BCP films^24^,^25^. Sivaniah *et al.* fabricated a perforated bicontinuous multilayer in sphere-forming BCP films, which were prepared through the osmotic shock with a selective solvent^26^. Most notably, a gyroid morphology has been explored and considered in the material science field to be the most attractive 3D nanostructure, because its inherent nanoscopic network provides a well-developed continuous pathway. For instance, Steiner, Snaith and coworkers used a uniform nanoporous template produced from a gyroid morphology, where the replicated metal-oxide materials in all interconnected channels could enhance the solar cell and electrochromic performances^27^,^28^. Ho *et al.* also reported the inorganic nanoporous gyroid structures templated from self-assembled BCPs, which enhanced photocatalytic efficiency by virtue of the increased porosity and surface area^29^. Despite vast potential uses of the interconnected and/or gyroid-like morphologies from self-assembled BCPs, the issues on the structural and mechanical stability have severely limited their practical utilization due to the low-molecular-weight properties in a weak segregation limit of BCPs.

To overcome this hurdle, we herein propose an implementable approach to the direct development of 3D bicontinuous gyroid nanostructure using strongly segregating high-molecular-weight BCP (*M*_*n*_ > 250 kg mol^−1^) of polystyrene-*b*-poly(methyl methacrylate) (PS-*b*-PMMA) to ensure the enhanced mechanical stability. The solvent-annealed BCP films have successfully produced target morphologies immediately after the fast evaporation of solvent, because the unique asymmetric composition (*ϕ*_*PS*_ = 0.640~0.660) of the high-molecular-weight BCPs could generate the morphological transition during the long-term stable SVA process with a neutral solvent, as schematically depicted in Fig. 1. Along with structural development, a well-defined gyroid structure displaying long-range order was finally achieved over a large area, after formation of a cylindrical structure at an early stage. Unlike other studies emphasizing the importance of 3D nanostructures for performance^24^,^25^,^26^,^27^,^28^,^29^,^30^,^31^, only in a high-molecular-weight BCP system could we find an approach to compare the morphology-dependent transport and filtration characteristics of two different nanostructures, i.e., those with hexagonal and gyroid structures from a single material. We further demonstrated an inverse skeletal GYR replica using a pristine GYR morphology as a sacrificial template, as shown in Fig. 1.

## Results and Discussion

**Figure 1:**
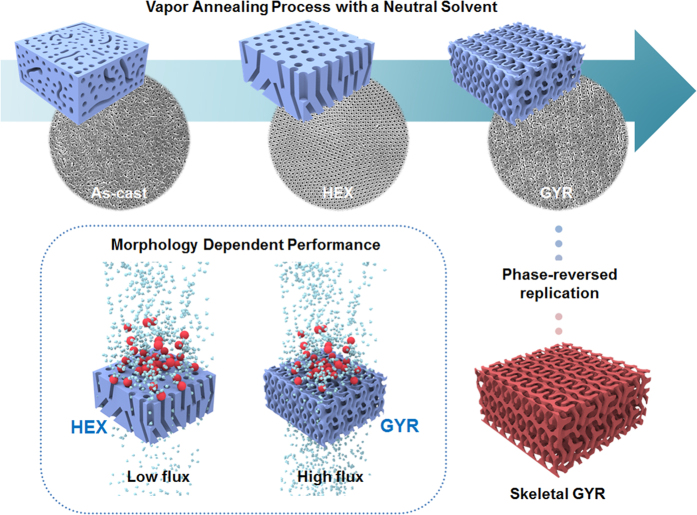
Major routes for producing nanoporous PS templates and further applications. Schematics of a morphological transition from the HEX to GYR structures during the SVA process, morphology-dependent performance in flux and ultrafiltration, and the inverse GYR replica pattern transferred from a GYR sacrificial template.

We first used an asymmetric PS-rich 278 kg mol^−1^ PS-*b*-PMMA for generating 3D bicontinuous complex nanostructure. A 0.5-μm-thick BCP film was prepared onto a standard Si substrate and subjected to the SVA process with tetrahydrofuran (THF) vapor as a neutral solvent for both blocks of PS-*b*-PMMA. A recently developed technique was exploited to regulate the solvent absorption and dewetting times of the swollen PS-*b*-PMMA films, where an optimized temperature gap between the chamber and bottom plate ensures a long-term stable SVA process that facilitates translational order of BCP to generate well-defined nanostructures^32^. Figure 1 shows the major routes for producing nanoporous PS templates, corresponding top-view scanning electron micrograph (SEM) images after the PMMA-removal process, and their further applications. This schematic represents that a morphological transition through a hexagonal (HEX) cylinder to a gyroid (GYR) morphology occurred during the time-controlled SVA process. Nanostructures in the films swollen during the SVA process were fixed as a result of fast evaporation of solvent. A poorly ordered as-cast film that was frozen due to a kinetic trap developed into more ordered morphologies when the BCP films absorbed THF vapor. Solvent-annealed BCP films at different periods of the SVA process were characterized by atomic force microscopy (AFM) using a phase mode (See Fig. S1 in Supplementary Information). The resulting images indicated that a HEX morphology (for 35 min) underwent the phase transition to a complex bicontinuous GYR structure during 100 min of the SVA process, after which a [211] GYR plane appeared at the surface with further annealing.

**Figure 2:**
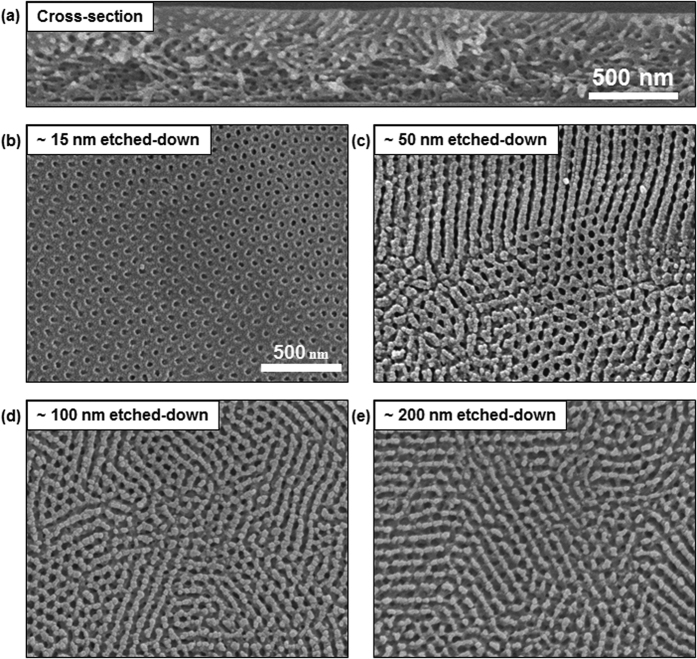
Structural analysis of HEX morphology. (**a**) Cross-sectional SEM image showing the HEX morphology obtained at 35-min of SVA process. The nanopores were initially oriented perpendicular to the surface and then randomly oriented in the interior of the films. (**b**–**e**) Top-view SEM images that were obtained during the surface etching process with an O_2_/Ar (5/1 in volume ratio) gas mixture, where the etched thicknesses of the films are indicated in the SEM images.

Figure 2 shows the SEM images of HEX morphology that was obtained at 35 min during the SVA process. A cross-sectional (Fig. 2a) SEM image indicated the nanopores to be initially perpendicular to the surface and then randomly oriented in the interior of the films, which was confirmed by top-view (Fig. 2b–e) SEM images obtained during the surface etching process with an O_2_/Ar (5/1 in volume ratio) gas mixture. The interfacial interactions between the neutral THF vapor and both blocks of PS-*b*-PMMA were relatively balanced, and the surface energies of the PS and PMMA blocks were very similar, resulting in the cylindrical microdomains oriented normal to the surface. This orientation, however, dissipated with distance from the surface, leading to the randomly oriented cylinders in the interior of the films, which is evidently shown in Fig. 2e by a mixed orientation of cylinders.

**Figure 3:**
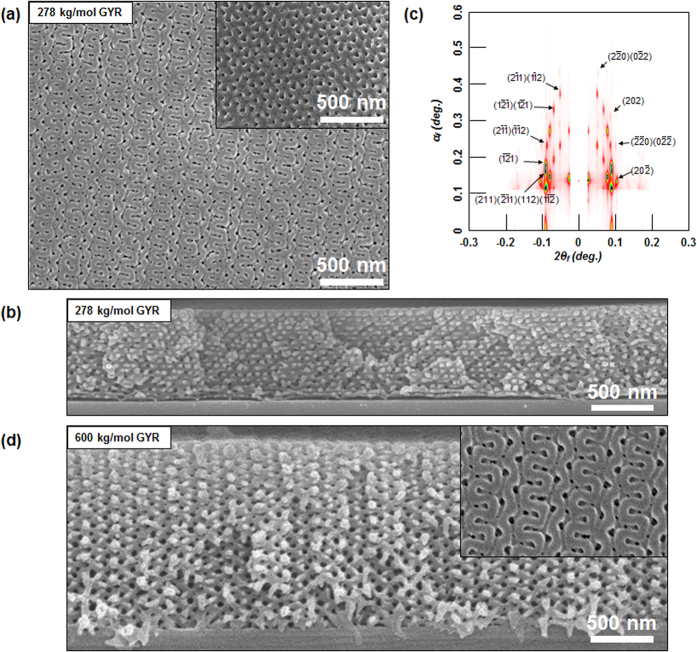
Structural analysis of GYR morphology. (**a**) Top-view SEM images showing the GYR structure of the 278 kg/mol PS-*b*-PMMA film, in which the PMMA block was selectively removed by UV irradiation and acetic acid rinsing. The images indicated a major type [211] plane and a minor type [111] plane (inset). (**b**) Cross-sectional SEM image showing a bicontinuous double GYR morphology across the entire thickness (278 kg/mol PS-*b*-PMMA film). (**c**) 2D GISAXS pattern revealing the GYR morphology (278 kg/mol PS-*b*-PMMA film). Characteristic peak indexes assigned in the pattern were evident, indicating the long-range order of the GYR structure and its interconnectivity. The contraction ratio was evaluated to be 0.28 based on a reflected point of *q*_*z*_ in the {202} plane (Fig. S2 in Supplementary Information), because the variation occurred vertically in a confined film geometry. (**d**) Cross-sectional and top-view inset SEM images showing a very large scale of the GYR morphology of the 600 kg/mol PS-*b*-PMMA film.

On the other hand, Fig. 3a–c shows the structural analyses of GYR morphology that was obtained at 100 min during the SVA process. The top-view (Fig. 3a) and cross-sectional (Fig. 3b) SEM images of the film subjected to the SVA process for 100 min revealed a bicontinuous double GYR morphology across the entire thickness. Here, the SEM images in Fig. 3a indicated a major type [211] plane and a minor type [111] plane (inset). This film structure was confirmed with grazing incidence small-angle X-ray scattering (GISAXS) measurements. In the scattering geometry, *α*_*f*_ and *2θ*_*f*_ are the exit angles of the X-ray beam along the *out-of-plane* scattering normal to the sample surface, and along the *in-plane* scattering normal to the incidence plane (or parallel to the sample surface), respectively, where *q* = *(4π/λ) sin θ*_*f*_ is the scattering vector. An incidence angle (*α*_*i*_) was set at 0.140°, which is above the critical angle (0.113°) for PS-*b*-PMMA films to trace the film morphology across the entire thickness. Figure 3c shows a 2D GISAXS pattern of GYR morphology. Characteristic peak indexes assigned in the pattern were evident, indicative of the long-range order of the GYR structure and its interconnectivity. Interestingly, this pattern was elongated in the *out-of-plane* direction, implying that the GYR structure with a cubic lattice during the SVA process contracted (was compressed) in the direction normal to the surface immediately upon evaporation of solvent. The contraction ratio was evaluated to be 0.28 based on a reflected point of *q*_*z*_ in the {202} plane (See Fig. S2 in Supplementary Information), because the variation occurred vertically in a confined film geometry. Similarly in an asymmetric 600 kg mol^−1^ PS-*b*-PMMA film with greater molecular weight, a very large scale of the GYR morphology was observed during a long-term stable SVA process, as shown in the cross-sectional and top-surface (inset) SEM images of Fig. 3d. The lattice spacing of the {220} plane based on the (20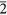) peak of 2D GISAXS patterns increased from 62 to 104 nm when the molecular weight of PS-*b*-PMMA was increased from 278 to 600 kg mol^−1^.

**Figure 4:**
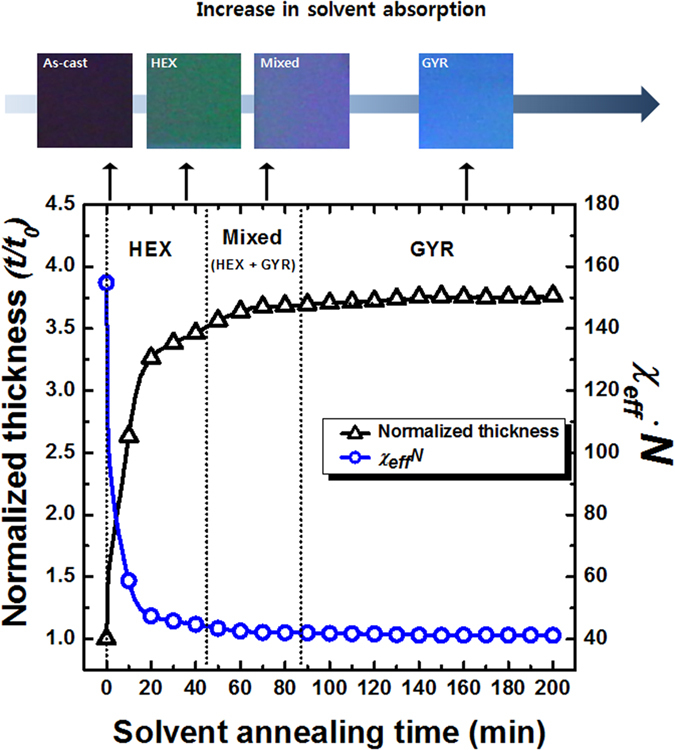
Solvent absorption behavior of the 278 kg/mol PS-*b*-PMMA film and *χ*_*eff*_* ·N* during the SVA process. The thickness of a swollen BCP film with a neutral solvent was normalized by the initial thickness (*t*_*o*_ = 500 nm). The effective interaction parameter was defined as *χ*_*eff*_ = *χ·Ф*, where *χ* = 0.0425 + 4.046/*T* and *Ф* = *t*_*o*_*/t*. The color change in the optical microscopy (OM) images on the top line of the figure reflects an increase in the film thickness during the SVA process. The morphological transitions are indicated by the dotted lines.

Figure 4 shows the solvent absorption behavior of the BCP film and *χ*_*eff ·*_*N* during the SVA process, which were measured *in situ* using a spectroscopic reflectometer. The thickness of a swollen BCP film with a neutral solvent was monitored, as shown by the normalized thickness (*t/t*_*o*_, *t*_*o*_ = 500 nm) as a function of SVA time. The effective interaction parameter, which accounts for the screening effects of the solvent molecules at the interfaces between the two blocks, can be defined as *χ*_*eff*_ = *χ·Ф*, where *χ* was approximated to be *χ* = 0.0425 + 4.046/*T* for a symmetric composition^33^, and the volume fraction (*Ф*) of BCP was calculated to be *Ф* = *t*_*o*_*/t*. The color change in the optical microscopy (OM) images on the top line of the figure reflects an increase in the film thickness during the SVA process. At an early stage of the SVA process, the film thickness increased rapidly to *t/t*_*o*_ ~ 3.2 and the *χ*_*eff ·*_*N* decreased from 154.8 to 48.4, as the morphology of the as-cast film transformed into an ordered HEX structure, as delineated by the dotted line in the figure. The appearance of the HEX structure was presumably attributed to fast initial absorption of solvent by the PS block at an early stage, although the saturated absorption ratio of THF to the PS and PMMA blocks was evaluated to be nearly neutral (or nonselective) for both blocks^19^.

Upon further increasing SVA time over 100 min, the *t/t*_*o*_ increased much more slowly and then plateaued at ~3.7, while the *χ*_*eff *·_*N* decreased much more slowly and then leveled off at a value of 42. The high *χ*_*eff* ·_*N* value, far above 10.5 at the order-to-disorder transition, indicated that during the SVA process the BCP film was still subject to a strong driving force to segregate into the two blocks, leading to the GYR structure displaying long-range order. When the solvent vapor was removed at a GYR morphology with *t/t*_*o*_ ~ 3.7 (100 min), the contraction ratio of the film thickness was measured to be 0.27; this value was identical to the contraction ratio of 0.28 evaluated from the GISAXS pattern (See Fig. S2 in Supplementary Information) within experimental error. However, it should be pointed out that the values of *t/t*_*o*_ were variable for the GYR morphology by controlling the solvent absorption of BCP films. For example, the GYR morphology was formed at *t/t*_*o*_ ~ 3.3 when the chamber temperature was decreased from 23 to 20 °C with respect to a constant bottom temperature of 30 °C (See Fig. S3 in Supplementary Information). In the case of the 600 kg mol^−1^ PS-*b*-PMMA film, a stable giant GYR morphology (Fig. 3d) was achieved with a *t/t*_*o*_ ~ 5 (at 420 min) corresponding to an *χ*_*eff* ·_*N* *~* 64 (See Fig. S4 in Supplementary Information). Hence, when using high-molecular-weight PS-*b*-PMMA, the development of the GYR from HEX structure in the strong segregation limit was enabled not only by increasing the chain mobility in highly entangled chain conformation but also by effectively decreasing the unfavorable interactions between the two blocks using a neutral solvent. Our study based on the swelling-controlled BCPs was in good agreement with the extended phase stability of the GYR structure determined by the self-consistent field theory (SCFT)^34^.

**Figure 5:**
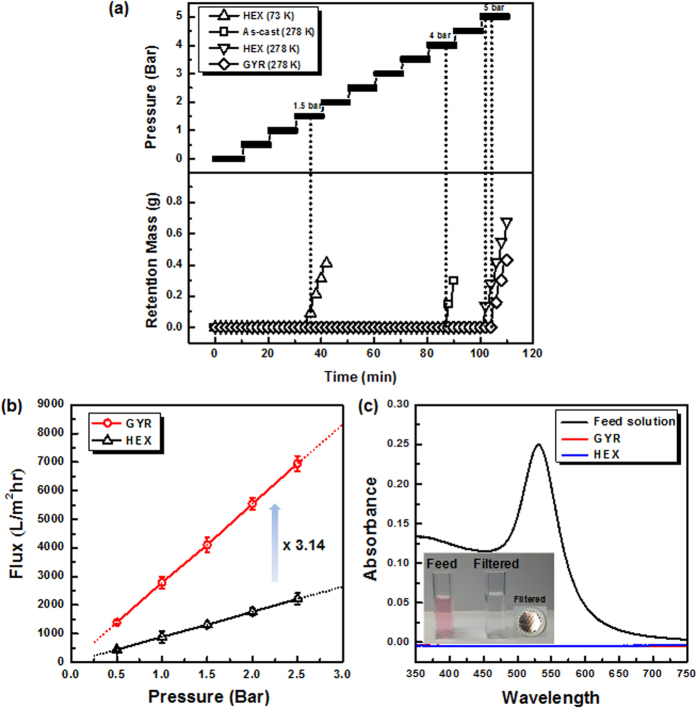
Mechanical stability and membrane performance of BCP films. (**a**) Retention mass as a function of hydrostatic pressure to determine burst pressure (*P*_*b*_) of the 278 kg/mol PS-*b*-PMMA films. The *P*_*b*_ was determined to be the pressure at which a mass of DI water was first detected to pass through the composite layers as the hydrostatic pressure was stepwisely increased from 0.5 to 5.5 bar by 0.5 bar steps every 10 min. (**b**) Water flux of nanoporous UF membranes with the HEX and GYR structures, where the BCP films obtained from swelling-controlled experiments were treated with the PMMA-removal process. (**c**) UV-Vis absorption spectra of the feed and filtrated solutions through the composite BCP membranes pressurized at 1 bar, indicating that the 60-nm NPs were effectively rejected by the two membranes.

To elaborate on the mechanical stability of the 278 kg mol^−1^ PS-*b*-PMMA films for membrane applications, the composite layers were prepared comprising a 0.5-μm-thick BCP film on a 150-μm-thick poly(ethersulfone) (PES, Sterlitech) supporting membrane with a pore diameter of 0.45 μm. Note that the PMMA block as a minor component has not yet been removed from the BCP films. The burst pressure (*P*_*b*_) was determined as the pressure at which a mass of deionized (DI) water was first detected after penetrating through the collapsed composite layers as the hydrostatic pressure onto the BCP film layer was stepwisely increased from 0.5 to 5.5 bar by 0.5 bar steps every 10 min. Figure 5a shows the retention mass as the hydrostatic pressure was increased, where a 73 kg/mol PS-*b*-PMMA film with a cylindrical morphology was used for comparison. The *P*_*b*_ of the high-molecular-weight BCP films was about 4~5 bar, much higher than the 1.5 bar determined for the 73 kg/mol BCP, confirming the significantly improved mechanical stability of the film with increasing the molecular weight of BCP. In addition, it was evident that the *P*_*b*_ in ordered structures was greater than that in the as-cast film, though it was rarely influenced by the morphological change in high-molecular-weight BCP films whether a HEX or a GYR structure.

Figure 5b shows the water permeability of nanoporous membranes with the HEX and GYR structures as a function of hydrostatic pressure applied from 0.5 to 2.5 bar, where the BCP films obtained from swelling-controlled experiments were treated with the PMMA-removal process in order to generate nanoporous channels in the PS matrix. To our surprise, the GYR membrane showed a flux of 2800 L/m^2^h at 1 bar, approximately 3 times greater than the flux through the HEX membrane (890 L/m^2^h at 1 bar). The flux (υ) values are associated with the structural parameters of the membranes according to the Hagen-Poiseuille relation,


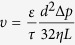


where *ε, τ, η* and *L* denote the void fraction, tortuosity of the porous structure, water viscosity (*η* = 1.002 mPa·sec at 20 °C), and length (or film thickness, *L* = 500 nm), respectively. The void fraction (*ε*) was determined to be 0.27 = 0.34 × 0.78 considering a PMMA volume fraction (*Ф*_*PMMA*_ = 0.34) multiplied by the porosity of 0.78 for the supporting membrane. A pore diameter (*d*) from the HEX structure was set to 51.6 nm for both membranes. The tortuosity (*τ*) values for the HEX and GYR membranes were calculated to be 17.9 and 5.7 from the Hagen-Poiseuille relation, respectively, which were quantitatively consistent with the superior flux performance in the nanoporous GYR structure due to the interconnected bicontinuity. This result demonstrated the morphological excellence of the GYR-structured membrane on the basis of these comparative results between the two nanoporous membranes made of a single BCP material. Even these water fluxes were much greater than that (195 L/m^2^h) of the conventional poly(ether sulfone) (PES) ultrafiltration (UF) membrane possibly due to the enhanced porosity of the interconnected nanopores^6^.

Next, a solution of Au nanoparticles (NPs) with an average diameter of 60 nm was used to measure the rejection efficiency of these NPs for ultrafiltration separation test, where the NP dispersed solution was prepared in DI water. Figure 5c shows UV-Vis absorption spectra of the feed and filtrated solutions through the composite BCP membranes pressurized at 1 bar. A maximum absorbance at 530 nm was characteristic of 60-nm Au NPs in the feed solution. When the solution was filtered through HEX and GYR membranes, the maximum absorbance disappeared completely irrespective of the morphology, indicating the creation of the uniform nanopores of BCP membranes prepared with swelling-controlled SVA experiments. The solution color associated with the Au NPs vanished after filtration, and the NPs were effectively rejected from both types of membranes, as also shown in the inset in Fig. 5c.

**Figure 6:**
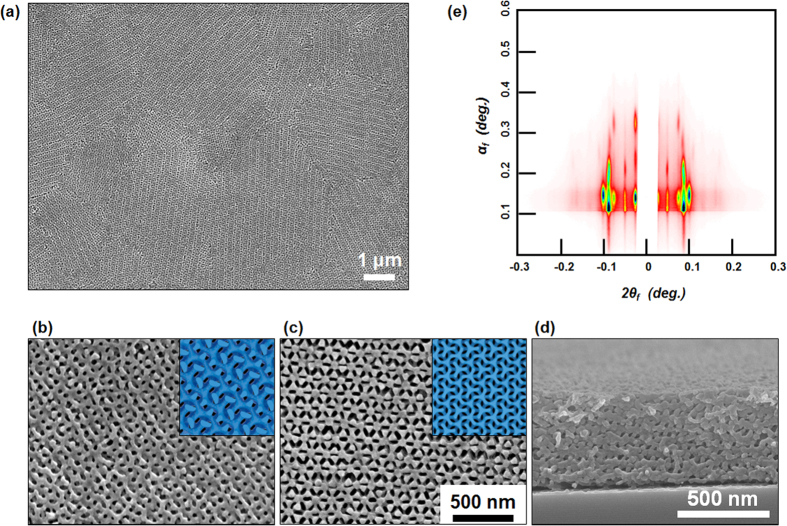
Inverse skeletal GYR replica and structural analysis. (**a**) Large-area SEM image showing an oxidized PDMS template inversely replicated from the nanoporous GYR template using the 278 kg/mol PS-*b*-PMMA film. Magnified top-view SEM images showing (**b**) a major type [211] plane and (**c**) a minor type [111] plane of the GYR replica. The images were consistent with schematics of the projection (insets). (**d**) Cross-sectional SEM image showing the uniform interconnectivity of the inverse GYR template across the entire thickness. (**e**) 2D GISAXS pattern revealing the characteristic peaks of the contracted GYR in the direction normal to the surface.

We further demonstrated a pattern transfer of a nanoporous GYR template obtained from the SVA process. A nanoporous PS template infiltrated with a PDMS pre-polymer was cured under vacuum, and followed by a treatment with O_2_ plasma to remove the template, leading to an inverse template of oxidized PDMS. Figure 6a displays a large-area SEM image of an oxidized PDMS template inversely replicated from the nanoporous GYR sacrificial template using the 278 kg mol^−1^ PS-*b*-PMMA film. The magnified top-view SEM images are shown in Fig. 6b,c with schematics of the projection (insets), corresponding to a major type [211] plane and a minor type [111] plane of the GYR replica, respectively. A cross-sectional SEM image, shown in Fig. 6d, exhibited the uniform interconnectivity of the inverse GYR structure across the entire thickness. This structural feature of the inverse skeletal GYR replica was confirmed by GISAXS measurements, as shown in Fig. 6e. Characteristic peak indexes in the GISAXS pattern were identical to those observed for the contracted GYR in the direction normal to the surface, although the intensity was relatively weak due to a decrease in the scattering contrast. The succesful structural replication of GYR structure might be applied to organic or inorganic hybrid materials, photonic crystals, energy harvesting materials for further application.

## Conclusion

We have developed a simple and robust route to producing a mechanically stable bicontinuous GYR membrane from high-molecular-weight PS-*b*-PMMA films with the unique asymmetric composition (*ϕ*_*PS*_ = 0.640~0.660). Here, a long-term stable SVA process enabled us to produce a well-defined GYR structure displaying long-range order as well as a HEX structure. The chain mobility was accelerated by effectively decreasing the unfavorable interactions between the two blocks using a neutral solvent, but the interfacial uniformity of nanostructures were attributed to the high *χ*_*eff* ·_*N* value still in the strong segregation limit. Besides the improved mechanical stability of the membranes, the nanoporous GYR membrane exhibited a three-fold greater flux than did the HEX membrane on account of the reduced tortuosity in the porous structure. We also demonstrated the versatility of the 3D nanoporous GYR template that was applied to an affordable material to reproduce an inverse skeletal replica of the GYR structure with its structure being uniformly interconnected. This result suggests its feasible structural tunability to the GYR structure, which would open the vast possible avenues applicable to other affordable materials.

## Methods

### Synthesis and sample preparation

Compositionally asymmetric PS-*b*-PMMAs were synthesized by sequential anionic polymerization of styrene (S) and methyl methacrylate (MMA) in tetrahydrofuran (THF) solvent; this reaction was performed at −78 °C in the presence of LiCl (high purity, Aldrich) under a purified argon environment using *sec*-butyllithium as an initiator. The number-averaged molecular weight (*M*_*n*_) was characterized by size-exclusion chromatography (SEC), and the dispersity (*Đ* = *M*_*w*_*/M*_*n*_) was narrower than 1.05. The PS volume fraction (*ϕ*_*PS*_) of 278 kg mol^−1^ and 600 kg mol^−1^ PS-*b*-PMMAs were determined to be 0.660 and 0.640, respectively, by ^1^H nuclear magnetic resonance (^1^H-NMR), based on the mass densities of the two components (1.05 and 1.184 g cm^−3^ for PS and PMMA, respectively). The PS-*b*-PMMA films were prepared by spin-coating in a standard Si wafer typically at 3500 rpm for 60 s using 5~6 wt% BCP solutions in toluene to set the film thickness at 0.5 μm. The film thicknesses were measured using a spectroscopic reflectometer (S-TRC-UV-NIR, Wonwoo Systems Co.).

Tetrahydrofuran (THF; high purity, Aldrich) was used as a neutral solvent for the SVA process with PS-*b*-PMMA films. A cylindrical brass chamber was devised to set the volume (V = 706.5 cm^3^) and surface area (S = 78.5 cm^2^) of solvent, where the solvent absorption of BCP films was precisely modulated by varying the annealing period under the condition of S/V = 0.111 cm^−1^. The chamber was completely sealed with Teflon cap and a Chemraz (Greene Tweed Co.) O-ring. A recently developed technique with an optimized temperature gap between the chamber and bottom plate was applied to the SVA process to regulate the solvent absorption and dewetting times of the swollen PS-*b*-PMMA films^32^. The chamber and bottom temperatures were set to 23 and 30 °C, respectively, for the 278 kg mol^−1^ PS-*b*-PMMA films, and to 43 and 48 °C, respectively, for the 600 kg mol^−1^ PS-*b*-PMMA films.

### Characterization of BCP films

Grazing-incidence small-angle X-ray scattering (GISAXS) experiments were performed at the 9A beam-line at Pohang Accelerator Laboratory (PAL), Korea. The typical operating conditions were set at a wavelength of 1.112 Å and a sample-to-detector distance of 6.48 m. The incidence angle (*α*_*i*_) was set at 0.080 to 0.140^o^, which were below and above the critical angle (0.113^o^) to probe the surface and entire film structures, respectively. 2D GISAXS patterns were recorded using a 2D detector (SX-165, Rayonix) positioned at the end of a vacuum guide tube with an exposure time of 10 s.

To examine the surface morphology of PS-*b*-PMMA films, atomic force microscopy (AFM; Dimension 3100, Veeco Digital Instrument Co.) was operated in a tapping mode. A standard silicon nitride probe was used at 3% offset below their resonance frequencies ranging from 250 to 350 kHz, where height and phase images were taken at a scanning speed of 7 μm/s. SEM images of dry-etched PS-*b*-PMMA films were measured with a field emission scanning electron microscope (FE-SEM; JSM-6701F, JEOL) under an accelerating voltage of 5.0 kV using a semi-in-lens detector. To enhance the phase contrast between the PS and PMMA blocks, asymmetric dry (or plasma) etching (VITA, Femto Sci.) mode was operated with an O_2_/Ar (5/1 in volume ratio) gas mixture under an RF power of 100 W at 150 mTorr and 18 sccm.

### Characterization of membrane performance

The PS-*b*-PMMA films were exposed to UV irradiation (*λ* = 254 nm) under vacuum for 3 h, and thoroughly rinsed with acetic acid for 1 h to selectively remove the PMMA block. The films were further cleansed with ethanol and DI water sequentially, and dried in a vacuum oven at room temperature. To fabricate a composite ultrafiltration (UF) membrane, the UV-etched, nanoporous BCP films were floated onto the surface of a 5 wt% HF solution, and transferred to the top of a 150-μm-thick, macroporous poly(ethersulfone) (PES) supporting membrane with an average pore diameter of 0.45 μm (Sterlitech). A home-made UF membrane cell was devised to measure the permeability of DI water and size selectivity at room temperature; this cell had a 10-ml working volume and an effective membrane area of 0.636 cm^2^. Water flux was measured with DI water at various pressures from 0.5 to 2.5 bar. Non-etched films were used to examine the mechanical strength by applying pressure from 0.5 to 5.5 bar. An Au NP solution with an average NP diameter of 60 nm (Sigma-Aldrich) was filtered through the nanoporous BCP membranes to characterize the size selectivity of the UF membranes, where the concentrations of Au NP in the feed and filtered solutions were analyzed by UV-Vis absorption spectroscopy.

### Pattern transfer from nanoporous BCP template

Polydimethylsiloxane (PDMS) precursor (Sylgard 184, Dow Corning Co.) was used to permeate the nanoporous BCP template, and then the filled template was cured at 60 °C under vacuum for 12 h. The cured composite film was exposed to plasma etching (VITA, Femto Sci.) with O_2_ under an RF power of 100 W at 150 mTorr and 20 sccm; this step removed the PS template efficiently, as a result, an inverse replica of the oxidized PDMS remained.

## Additional Information

**How to cite this article**: Park, S. *et al.* Giant Gyroid and Templates from High-Molecular-Weight Block Copolymer Self-assembly. *Sci. Rep.* **6**, 36326; doi: 10.1038/srep36326 (2016).

**Publisher’s note:** Springer Nature remains neutral with regard to jurisdictional claims in published maps and institutional affiliations.

## Supplementary Material

Supplementary Information
