# Supplementary material for: Giant Gyroid and Templates from High-Molecular-Weight Block Copolymer Self-assembly
Source: Sci Rep. 2016 Nov 3;6:36326. doi: 10.1038/srep36326 (PMC5093756; doi:10.1038/srep36326)
Supplement: Supplementary Information [file srep36326-s1.doc]

**Supplementary Information**

**Giant Gyroid and Templates from High-Molecular-Weight Block Copolymer Self-assembly**

Sungmin Park^1^, Yeongsik Kim^1^, Hyungju Ahn^2^, Jong Hak Kim^1^, Pil J. Yoo^3,*^ and Du Yeol Ryu^1,*^

^1^Department of Chemical and Biomolecular Engineering, Yonsei University, Seoul 03722, Korea.

^2^Pohang Accelerator Laboratory, Pohang University of Science and Technology, Pohang 37673, Korea

^3^School of Chemical Engineering and SKKU Advanced Institute of Nanotechnology (SAINT), Sungkyunkwan University (SKKU), Suwon 16419, Korea.

Correspondence and requests for materials should be addressed to P.J.Y. (email: pjyoo@skku.edu) or to D.Y.R. (email: dyryu@yonsei.ac.kr).


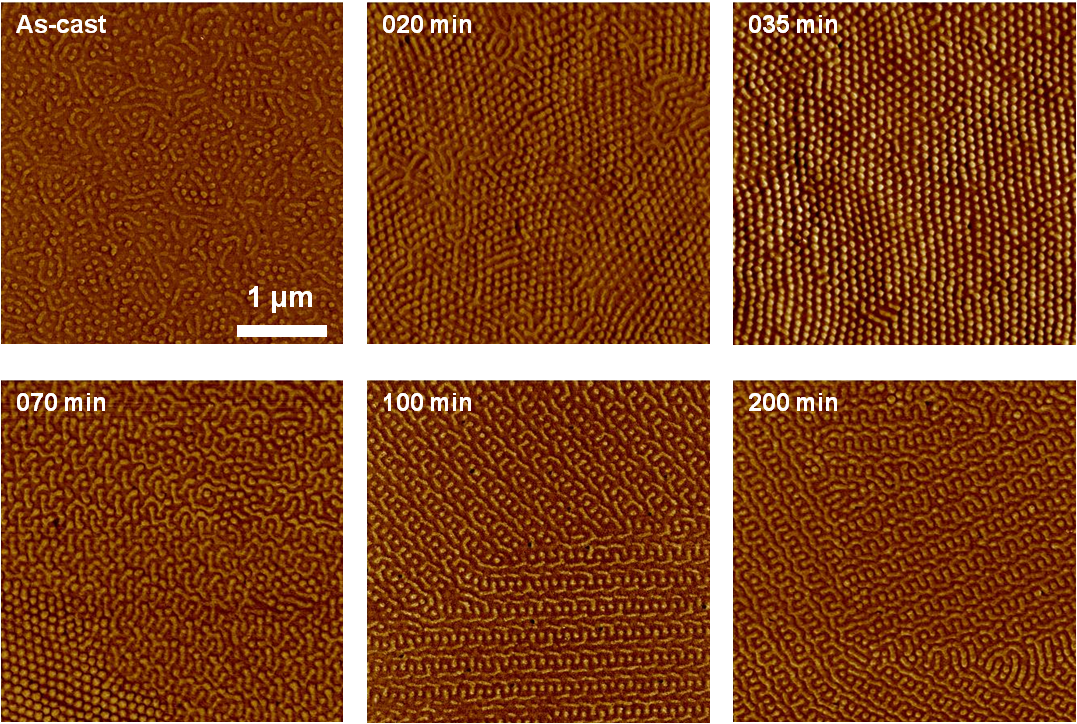


Figure S1. Structural development on the surface during the SVA process. AFM phase images of 0.5-μm-thick PS-*b*-PMMA films showing a phase transition from a HEX (for 35 min) to a complex bicontinuous GYR structure by 100 min of the SVA process, in which a [211] GYR plane appeared at the surface with futher annealing time.


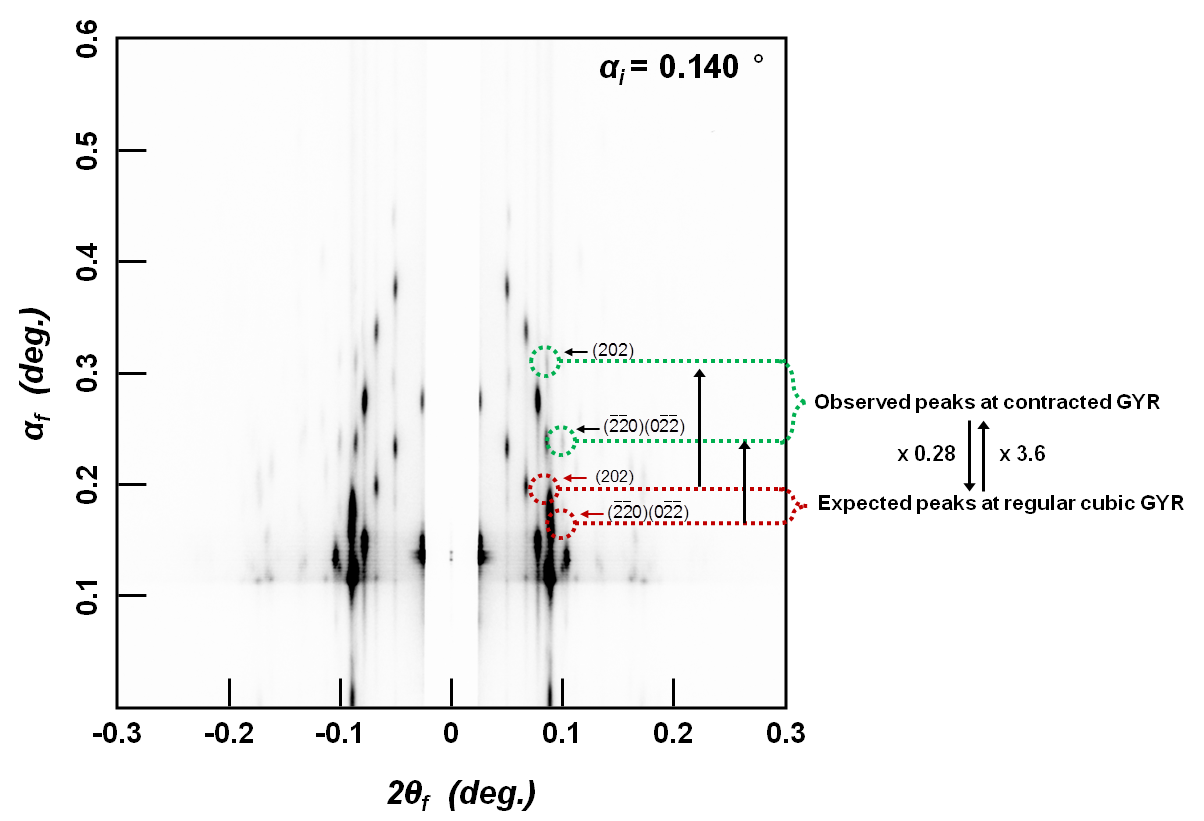


Figure S2. Contraction analysis based on 2D GISAXS pattern of the GYR morphology. The elongated pattern to *out-of-plane* direction indicated that the GYR structure was formed with a cubic lattice during the SVA process and contracted (was compressed) in the direction normal to the surface immediately upon evaporation of solvent. The contraction ratio was evaluated to be 0.28 based on a reflected point of *q_z_* in the {202} plane, because the variation occurred vertically in a confined film geometry. Alternatively, the regular GYR structure was formed when the 278 kg/mol PS-*b*-PMMA film swelled 3.6 times during the SVA process.


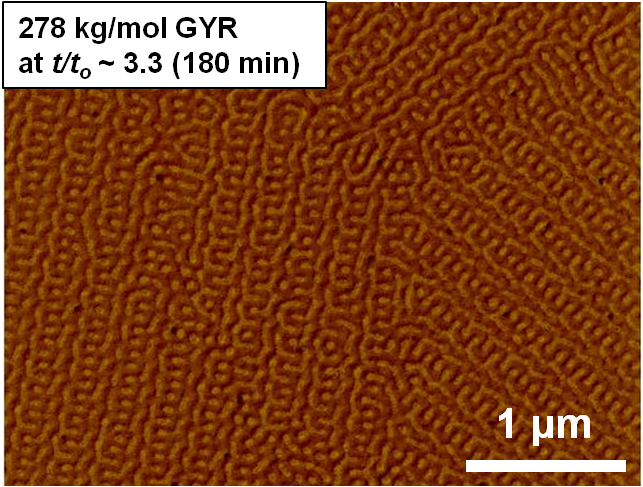


Figure S3. AFM phase image of GYR structure ([211] plane) for 278 kg/mol PS-*b*-PMMA film obtained at the reduced *t/t_o_* ~ 3.3. A 0.5-μm-thick PS-*b*-PMMA film was subjected to the modulated SVA process, where the chamber temperature was set to 20 ^o^C with respect to a constant bottom temperature of 30 ^o^C. Note that this chamber temperature was decreased by 3 ^o^C from 23 ^o^C in order to regulate the solvent absorption and dewetting times of the swollen PS-*b*-PMMA film.


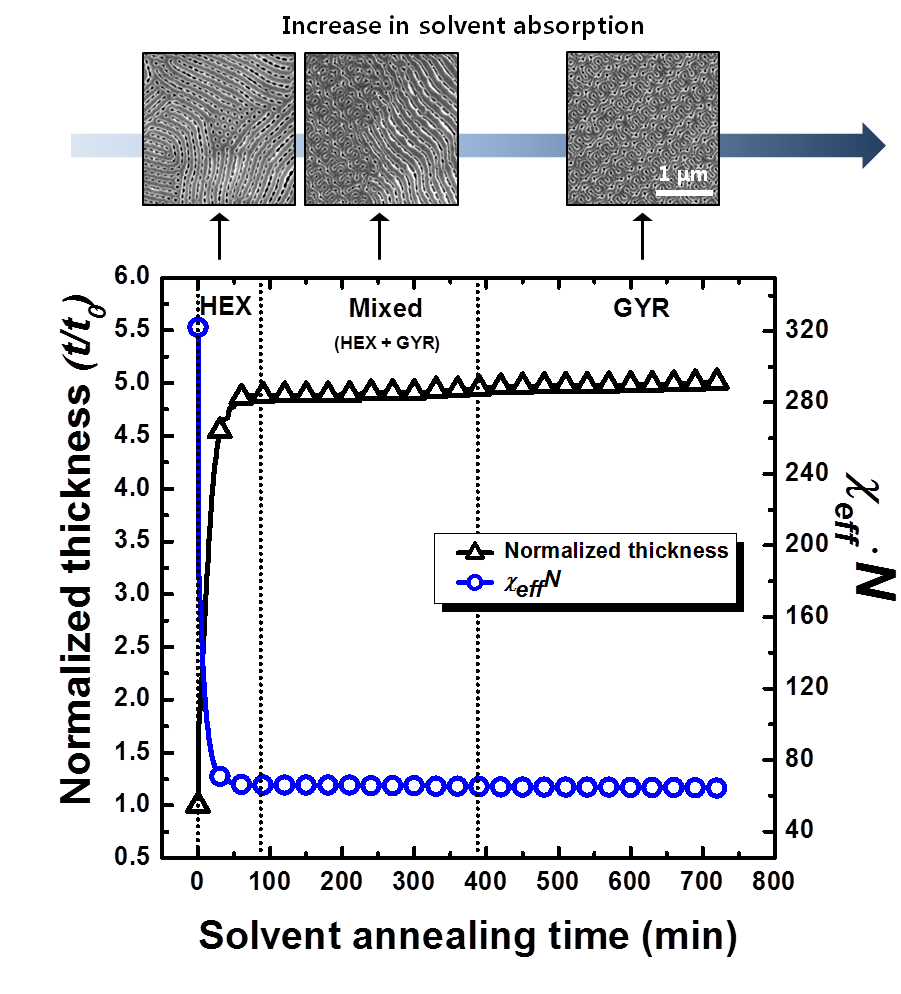


**Figure S4. Solvent absorption behavior of the 600 kg/mol PS-*b*-PMMA film and *χ_eff·_N* during the SVA process.** The thickness of a swollen BCP film with a neutral solvent was normalized by the initial thickness (*t_o_* = 1000 nm). The effective interaction parameter was defined as *χ_eff_ = χ·Ф*, where *χ* = 0.0425+4.046/*T* and *Ф = t_o_/t*. The top-view SEM images on the top line indicated the morphological transitions through the HEX to GYR structures in a very large scale during the time-controlled SVA process.
